# Yellow Fever

**Published:** 1876-11

**Authors:** 


### Editorial.

### YELLOW FEVER.

With sadness of heart we approach the consideration of this subject. Our sister cities of Savannah and Brunswick are now draped in the emblems of deep sorrow at the loss of hundreds of their noble, couragous citizens. Many were from home, at up-country summer resorts, when the pestilence commenced its ravages, and others, not a few, fled, panic-stricken, on the approach of the dread malady. Thus, food for the destroyer was lessened perhaps more than half. Lamentable indeed is it that all could not flee the destroyer. Those already sick and otherwise unable to escape the threatened danger, could not be forsaken in their distress, and many noble lives were sacrificed in affording aid to their suffering neighbors. Physicians, apothecaries, policemen, and other valient citizens, braved all danger in the sacred cause of humanity. At these sad reflections, the joy of our centennial jubilee "has been turned into mourning" throughout the country.

Scourges of this kind are of almost yearly occurrence on large rivers and the seacoast of the South, and as medical journalists we feel called upon by the subjects of this pestilence to urge the profession to institute more vigorous and thorough investigation as to the cause and nature of this disease. Humanity demands this of a learned and noble profession.

In our efforts to protect communities from this fell destroyer, it is not sufficient that we promise safety on the adoption of quarantine systems, however strictly enforced. This promised protection has doubtless lured thousands of valuable citizens into the fatal jaws of the monster. We should not longer rely on that which has so often deceived us.

The idea too generally prevails that yellow fever is contagious, or, at any rate, can be in some way communicated from one person to another; and when the advocates of this communication theory are met by the fact that many flee the infected district after having received the poison, and die of the disease without communicating it, they tell us there must be a nidus or nucleus before propagation by personal contact can occur. So common is this notion of contagion, that Dr. Greensville Dowell, of Texas, in his treatise on yellow fever, expresses great surprise that the people of San Antone did not contract the disease from yellow fever refugees stopping among them. Medical theorists compare a city, the subject of this disease, to tinder, ready to be lighted by a case landed from a vessel. Truly, the nucleus, nidus, tinder, or poison should engage attention; not, however, to guard it against inflammable cause from abroad, but to prevent its accumulation, its manufacture, by a change of conditions at home. If the poison be suffered to generate in sufficient quantity, and the people be exposed to it, the flame may certainly be expected, even without a foreign match to ignite.

Whether or not the morbific agent consists of ordinary malaria, certain it is that the conditions and circumstances which lead to the production of yellow fever are similar, in many respects, to those which give rise to remittent and intermittent fever. The most essential of these are, high temperature during the overflow of marshes or swamps by streams receiving the washings of cultivated lands; the still water of such ponds and marshes gradually subsiding in very hot and dry weather. The malignancy of yellow fever, and difference, in other respects, between it and simple cases of remittent fever, are not more striking than between the different forms of acknowledged malarial fever. Congestive or malignant intermittent and hæmaturial fevers are as unlike the simple forms of malarial fever, and are attended by mortality equal to that of the fever under consideration. Indeed, hæmaturial fever is perhaps even more fatal, and resembles yellow fever very much in the icterus hue and passive hemorrhage which attend it.

Again, it is known that in epidemics of yellow fever there are mild cases which, in general symptoms and termination, do not differ from ordinary bilious or remittent fever, while others are clearly intermittent, observing quotidian or tertian periodicity. This character of the disease was witnessed by Dr. L. D. Ford during the epidemic of 1839, in Augusta. Of three cases of yellow fever, attended by the writer, about the year 1845, and contracted in Panama by young men *en route* from California, one was malignant, and died with black vomit; another passed through the usual course of bilious fever for a week, and recovered; the third case was distinctly intermittent, and did not differ in any essential particular from ordinary *chills*, except that it yielded less readily to treatment than cases contracted around the mill-ponds of Georgia.

With the view of protection against this fatal malady in future, the State Board of Health will certainly inspect the surroundings of Savannah and Brunswick, if they have not already done so. Should marshes or pools be found, which had been flooded during the summer by the overflow of fresh water streams, the presumption is that the *causus morbi* had some connection with this condition. This view would be greatly strengthened by facts going to show that those most exposed to emanations from such location were the greatest sufferers. It is not always the case that the nearest inhabitants to such infected district suffer most. Elevated positions, even at the distance of a mile or more, are subject, unless obstructions, such as walls, trees with thick foliage, etc., should intervene.

